# Association Between Autonomic Symptoms and the Choroidal Vascularity Index in Fibromyalgia Patients

**DOI:** 10.3390/medicina62040748

**Published:** 2026-04-13

**Authors:** Dilara Ekici Zincirci, İrem Nur Yılmaz, Sevgi Atar, Esma Demirhan, İmran Arkan Emre, Gamze Karataş, Mehmet Zincirci, Demet Ferahman, Ömer Kuru

**Affiliations:** 1 Department of Physical Medicine and Rehabilitation, Prof. Dr. Cemil Taşcıoğlu City Hospital, Istanbul 34384, Turkey; 2 Department of Ophtalmology, Reşadiye State Hospital, Tokat 60500, Turkey; 3 Department of Ophtalmology, Prof. Dr. Cemil Taşcıoğlu City Hospital, Istanbul 34384, Turkey; 4 Department of Algology, Medical Faculty, Istanbul University, Istanbul 34080, Turkey; 5 Department of Physical Medicine and Rehabilitation, Dr. Sadi Konuk Bakırköy Training and Research Hospital, Istanbul 34140, Turkey

**Keywords:** autonomic nervous system, choroid, fibromyalgia, optical coherence tomography

## Abstract

*Background and Objectives*: Fibromyalgia syndrome (FMS) is frequently accompanied by autonomic symptoms and autonomic dysregulation, which may influence ocular blood flow regulation. The choroid is a densely vascular, autonomically innervated tissue, and optical coherence tomography (OCT)-derived markers have been used to explore potential ocular microvascular changes in FMS, with inconsistent findings. The choroidal vascularity index (CVI), defined as the proportion of luminal area within the total choroidal area, has been proposed as a potentially more robust marker of choroidal vascular status than thickness alone. We aimed to compare CVI and choroidal thickness between patients with FMS and healthy controls and examine the association between autonomic symptom burden and CVI in FMS. *Materials and Methods*: This single-centre observational cross-sectional case–control study enrolled adults aged 18–65 years. Swept-source OCT was performed; low-quality scans were excluded, and only right eyes were analysed. CVI, subfoveal maximum and mean choroidal thickness were obtained using an artificial intelligence-assisted analysis platform. Autonomic symptom burden, fibromyalgia impact, and central sensitization-related symptoms were assessed using the Composite Autonomic Symptom Score-31 (COMPASS-31), the Revised Fibromyalgia Impact Questionnaire (FIQ-R), and the Central Sensitization Inventory (CSI), respectively. Group comparisons, Spearman correlations, and multivariable linear regression were performed. *Results*: COMPASS-31, FIQ-R, and CSI scores were higher in the FMS group (all *p* < 0.001). CVI and choroidal thickness did not differ significantly between groups (CVI *p* = 0.124; maximum thickness *p* = 0.136; mean thickness *p* = 0.097). CVI was not correlated with COMPASS-31, FIQ-R, or CSI within either group. In adjusted models, age was independently associated with CVI (*p* < 0.001), whereas FMS status and COMPASS-31 total score were not. *Conclusions*: CVI and choroidal thickness were similar in FMS and controls, and CVI was not associated with self-reported autonomic symptom burden in FMS. Studies incorporating objective autonomic testing and dynamic vascular imaging paradigms are warranted.

## 1. Introduction

Fibromyalgia syndrome (FMS) is a chronic pain disorder characterised by widespread musculoskeletal pain with fatigue, sleep disturbance, cognitive impairment, and other somatic symptoms. It affects 2–4% of the global population [1]. Although the pathophysiology remains unclear, proposed mechanisms include central sensitisation and altered pain processing, hypothalamic–pituitary–adrenal axis dysregulation, and, in some patients, small fibre neuropathy [2]. Recent studies increasingly implicate autonomic nervous system dysfunction, reporting increased sympathetic activity, decreased parasympathetic activity, and disturbed sympathovagal balance in FMS [3,4]. This dysregulation may contribute to symptom burden and the syndrome’s multisystem presentation [5], supporting a potential role in its pathophysiology.

The choroid is the highly vascular layer located between the retinal pigment epithelium and the sclera (lamina fusca). Owing to its exceptionally high blood flow, it plays a central role in regulating ocular perfusion [6]. The choroid is richly innervated by parasympathetic and sympathetic fibres [7]; therefore, systemic haemodynamic changes and autonomic nervous system dysregulation can influence choroidal perfusion and thickness [8,9,10]. Optical coherence tomography (OCT) provides non-invasive, high-resolution cross-sectional imaging of the retina and choroid. However, total choroidal thickness—the most commonly reported metric—does not distinguish stromal from luminal components. The choroidal vascularity index (CVI), defined as the ratio of choroidal vascular area to total choroidal area, enables compartmental assessment and may mitigate limitations of thickness-only evaluation [11]. Its association with ocular microvascular changes in systemic diseases supports CVI as a candidate biomarker in clinical practice [6].

Central and autonomic nervous system dysregulation in FMS has been proposed to influence ocular microvascular structure and neuroretinal layers [12]. While some studies have shown thinning of the retinal nerve fibre layer and decreased choroidal thickness in FMS patients [13,14,15], others have not detected any significant changes in choroidal thickness [16,17]. Sevimli et al. reported higher CVI in patients with FMS than in healthy controls [18]. In contrast, Öztürk et al. and Talu Erten et al. observed no between-group differences in choroidal thickness or choroidal capillary density [16,19]. These inconsistencies may reflect methodological heterogeneity, including variability in imaging protocols and observer-dependent steps (e.g., manual segmentation and boundary delineation), which can increase intra- and inter-observer measurement error.

Most studies on ocular neurovascular changes in FMS have focused on associations between disease severity and retinal or choroidal metrics. In contrast, relatively few studies have examined the relationship between autonomic dysregulation and choroidal vascular structure. Accordingly, we investigated the association between autonomic symptom burden and CVI in patients with FMS. We hypothesised that higher autonomic symptom burden would be associated with altered CVI. To improve measurement standardisation and minimise observer-related variability, choroidal area analysis was performed using an automated artificial intelligence-based tool.

## 2. Materials and Methods

### 2.1. Study Design and Setting

This single-centre, observational, cross-sectional case–control study assessed the association between autonomic nervous system dysfunction and CVI in patients with FMS, compared with age- and sex-matched healthy controls.

Written informed consent was obtained from all participants. The study was conducted in accordance with the World Medical Association Declaration of Helsinki, as revised in October 2024. Ethical approval was obtained from the Medipol University Non-Interventional Clinical Research Ethics Committee (Approval No. 1231; 16 October 2025).

Participant recruitment was conducted between October 2025 and December 2025. After completion of recruitment and data collection, the study was registered retrospectively at ClinicalTrials.gov (Identifier: NCT07345546). As this study was designed as an observational cross-sectional case–control study rather than a prospective interventional trial, prior registration had not been planned; however, retrospective registration was performed to enhance transparency and provide a publicly accessible record of the study.

### 2.2. Study Population

Women and men aged 18 to 65 years were recruited prospectively between October and December 2025 from the outpatient Physical Medicine and Rehabilitation clinic at Prof. Dr. Cemil Taşcıoğlu City Hospital. Participants were allocated to the FMS or control group after eligibility screening and clinical assessment.

#### 2.2.1. Fibromyalgia Group

Patients in the FMS group fulfilled the 2016 revised American College of Rheumatology (ACR) criteria. Generalised pain was defined as pain in at least four of five body regions (left upper, right upper, left lower, right lower, and axial), with symptoms present for at least three months. Classification was based on the Widespread Pain Index (WPI; range 0 to 19) and the Symptom Severity Scale (SSS), which rates fatigue, unrefreshing sleep, and cognitive symptoms during the preceding week (each scored 0 to 3). Headache, lower abdominal pain or cramps, and depression were recorded as present or absent. Fibromyalgia was defined as WPI ≥ 7 with SSS ≥ 5, or WPI 4 to 6 with SSS ≥ 9, in line with the 2016 ACR framework. When available, the fibromyalgia severity score was calculated as WPI + SSS. Healthy control participants were recruited from the same outpatient clinic and matched with FMS patients based on age and sex. The controls had no history of FMS, chronic widespread pain, or systemic conditions known to affect choroidal structure or ocular microcirculation.

#### 2.2.2. Control Group

Healthy controls were recruited from the same outpatient clinic and matched to the FMS group by age and sex. Controls had no history of FMS, chronic widespread pain, or systemic conditions known to affect choroidal structure or ocular microcirculation.

#### 2.2.3. Exclusion Criteria

To support reliable OCT acquisition and reduce confounding, participants were excluded for ocular conditions likely to affect image quality or choroidal measurements. These included ocular media opacity (e.g., cataract), glaucoma, uveitis, optic neuropathy, significant ocular surface disease, and retinal or macular disorders, including retinal vascular pathology (e.g., diabetic retinopathy, retinal vein occlusion) and macular degeneration or dystrophy. Participants were also excluded if they had prior ocular surgery, recent ocular or head trauma, best-corrected visual acuity worse than 20/20, intraocular pressure > 21 mmHg, or current use of topical ophthalmic medications. To minimise the influence of ocular biometry, refractive error exceeding ±1.00 dioptres spherical equivalent was an exclusion; clinically significant astigmatism likely to affect choroidal measurements was also excluded.

Systemic exclusions comprised diabetes mellitus, hypertension, hyperlipidaemia, thyroid disorders, cardiovascular disease, inflammatory rheumatic or autoimmune disease (e.g., rheumatoid arthritis, vasculitis), polyneuropathy, neurodegenerative disorders, and other systemic conditions that could influence vascular parameters. Heavy smoking (>20 cigarettes/day), alcohol or substance dependence, and habitual high caffeine intake (>2.5 cups/day) were also exclusion criteria. To minimise treatment-related effects on symptom burden and autonomic measures, patients with FMS receiving fibromyalgia-specific pharmacological treatment at enrolment were excluded.

Eligibility was confirmed through clinical evaluation and review of hospital electronic medical records. Routine laboratory results were reviewed to exclude systemic conditions inconsistent with study eligibility.

### 2.3. Ophthalmological Examination

All participants underwent a standardised ophthalmological assessment, including objective refraction using an autorefractometer, intraocular pressure measurement by non-contact tonometry, and visual acuity evaluation. Anterior segment and fundus examinations were performed with slit-lamp biomicroscopy. Posterior segment examination was performed after pupil dilation when clinically indicated.

### 2.4. Outcome Measurements

#### 2.4.1. OCT Acquisition and Choroidal Measurements

Choroidal imaging was performed using swept-source optical coherence tomography (SS- OCT) with the DRI Triton^®^ device (Topcon Corporation, Tokyo, Japan). The axial resolution of the SS-OCT device (DRI Triton, Topcon) is approximately 8 µm, with a transverse resolution of approximately 20 µm, according to the manufacturer’s specifications. For each participant, a macular volumetric scan covering a 6 × 6 mm area centred on the fovea was acquired using the same protocol. To minimise diurnal variation in choroidal parameters, all scans were obtained in the morning (09:00–11:00) under standardised conditions and without pharmacologic mydriasis. During acquisition, participants were asked to maintain fixation on the internal target. Image quality was evaluated using the device signal quality score; scans with a score <6/10 or with motion artefacts were excluded. To avoid inter-eye correlation, only the right eye of each participant was included in the analysis.

**Figure 1:**
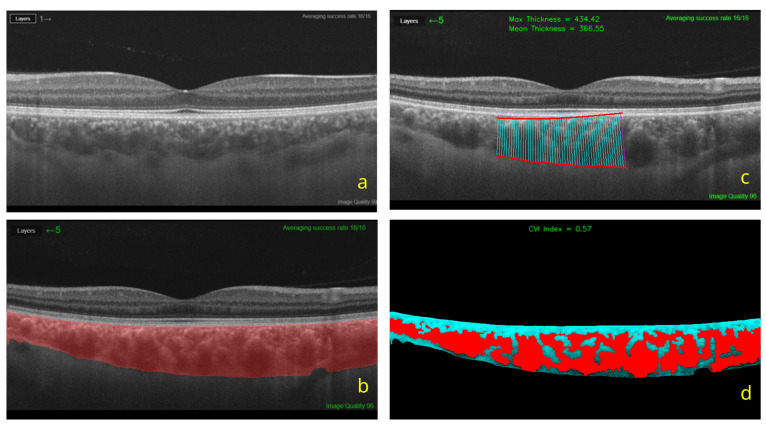
Artificial intelligence-assisted choroidal segmentation and calculation of choroidal thickness and CVI. (**a**) Original SS-OCT image. (**b**) Automated detection of the choroidal boundaries. (**c**) Definition of the region of interest for choroidal thickness measurement. (**d**) Binarized image used for CVI calculation, where the luminal (vascular) area is shown in red, and the stromal area is shown in turquoise.

To reduce observer-related variability in choroidal thickness and CVI estimation, choroidal segmentation and area-based metrics were derived using an artificial intelligence, deep learning-based platform (ChoroidAI; www.choroidometer.com, accessed on 1 December 2025). SS-OCT images were first screened independently by two experienced investigators (GK and IAE), who were blinded to group allocation and clinical information. Eligible images were then uploaded using a standardised workflow, and automated segmentation was used to obtain subfoveal (maximum) choroidal thickness, mean choroidal thickness, and CVI [20]. The region of interest was defined as the subfoveal choroidal area within the 6 × 6 mm macular scan centred on the fovea. CVI was calculated as the ratio of luminal area to total choroidal area within the segmented region of interest (Figure 1).

To further minimise observer-related variability, all segmentation procedures were primarily performed using an automated deep learning-based algorithm, reducing the need for manual boundary delineation. Independent review by two masked investigators ensured quality control, and any discrepancies were resolved by consensus. All images were anonymised prior to upload to ensure confidentiality. Outputs were recorded for subsequent statistical analyses.

#### 2.4.2. Composite Autonomic Symptom Score-31 (COMPASS-31)

Autonomic symptom burden was assessed using the COMPASS-31, a 31-item self-report questionnaire developed in 2012 that covers six domains: orthostatic intolerance, vasomotor, secretomotor, gastrointestinal, bladder, and pupillomotor symptoms. Using the standard weighted scoring algorithm, the total score ranges from 0 to 100, with higher scores indicating greater autonomic symptom burden [21]. The Turkish version has demonstrated validity and reliability in general populations [22] and specifically in patients with FMS [23].

#### 2.4.3. Revised Fibromyalgia Impact Questionnaire (FIQ-R) Score

Fibromyalgia-related disease impact was assessed using the FIQ-R. The FIQ-R consists of 21 items across three domains (function, overall impact, and symptoms) and yields a total score from 0 to 100, with higher scores indicating greater overall impact of fibromyalgia on daily life [24]. The Turkish version has demonstrated validity and reliability [25].

#### 2.4.4. Central Sensitization Inventory (CSI)

Central sensitization-related symptom burden was assessed using the CSI, a 25-item self-report scale scored 0–4 per item, producing a total score of 0–100, where higher scores indicate a higher burden of central sensitization-associated symptoms. The validity and reliability of the Turkish version were established by Keleş and colleagues [26].

### 2.5. Sample Size Calculation and Statistical Analysis

An a priori sample size calculation was performed for the primary comparison of CVI between the fibromyalgia and control groups. Based on an effect size estimate from prior literature (Cohen’s d = 1.0), a two-tailed α of 0.05 and 80% power (1 − β) yielded a minimum required sample of 27 participants per group [18]. The calculation was performed using G*Power version 3.1.9.7 (Heinrich Heine University, Düsseldorf, Germany). To account for potential data loss (e.g., insufficient OCT image quality), we aimed to recruit at least 30 participants per group. As an additional sensitivity check, given that d = 1.0 may be optimistic for OCT outcomes, we estimated the minimum detectable standardised effect size for the achieved sample (*n* = 42 and *n* = 38; two-sided α = 0.05), which was approximately d ≈ 0.64 for 80% power (≈0.73 for 90% power).

Statistical analyses were performed using IBM SPSS Statistics version 27.0 (IBM Corp., Armonk, NY, USA). Normality of continuous variables was assessed using the Shapiro–Wilk test. Continuous variables are presented as mean ± standard deviation (SD), and categorical variables as *n* (%). Between-group comparisons were performed using the independent-samples t test for normally distributed continuous variables and the Mann–Whitney U test for non-normally distributed continuous variables. Categorical variables were compared using the chi-square test or Fisher’s exact test, as appropriate. ES for between-group comparisons of continuous variables were reported as Hedges’ g (Fibromyalgia–Control) with 95% CIs; *p*-values were obtained from independent-samples *t*-test or Mann–Whitney U test according to distributional assumptions.

Associations between CVI and clinical measures (COMPASS-31, FIQ-R, and CSI) were examined within each group using Spearman’s rank correlation coefficients. To further evaluate independent associations and account for potential confounding, multivariable linear regression analyses were performed with CVI as the dependent variable. In the full sample, the primary regression model assessed the independent association between study group (FMS vs. control) and CVI after adjustment for age, sex, and body mass index (BMI) (Model A). Sensitivity analyses were then conducted by additionally adjusting this base model for smoking status (Model B), educational level (Model C), and occupational status (Model D), each entered separately. Within the FMS group, an additional multivariable linear regression model was constructed to examine the independent association between COMPASS-31 total score and CVI after adjustment for age, sex, and BMI. Regression coefficients (B), 95% confidence intervals (CIs), and *p* values were reported. Collinearity was assessed using tolerance and variance inflation factor diagnostics. A two-sided *p* value < 0.05 was considered statistically significant.

## 3. Results

A total of 80 participants were included (42 FMS and 38 controls) (Table 1). The groups were comparable in age (*p* = 0.463), sex distribution (*p* = 0.149), BMI (*p* = 0.128), and smoking status (*p* = 0.052). Educational level and occupational status differed significantly between groups (both *p* < 0.001).

Clinical outcomes are summarized in Table 2. Compared with controls, patients with FMS demonstrated significantly higher FIQ-R (*p* < 0.001; ES 0.80, 95% CI 0.70–0.86), CSI (*p* < 0.001; ES 0.76, 95% CI 0.65–0.84), and COMPASS-31 total scores (*p* < 0.001; ES 0.73, 95% CI 0.60–0.82). COMPASS-31 domain subscores were also significantly higher in the FMS group (orthostatic intolerance, vasomotor, secretomotor, gastrointestinal, bladder, and pupillomotor; all *p* ≤ 0.002), with effect sizes ranging from 0.35 to 0.63 (Table 2).

**Figure 2:**
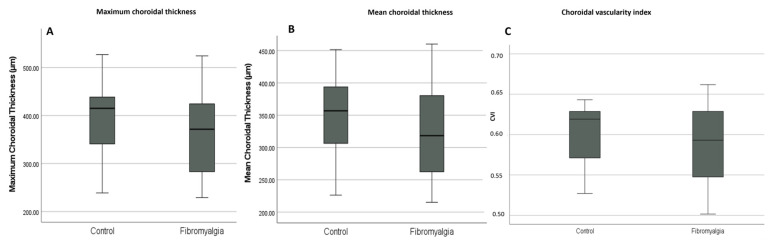
Distribution of choroidal parameters in the fibromyalgia and control groups. Boxplots show the distribution of (**A**) maximum choroidal thickness, (**B**) mean choroidal thickness, and (**C**) choroidal vascularity index (CVI) in the two study groups. The central line indicates the median, the box represents the interquartile range, and the whiskers indicate the spread of the non-outlier values.

OCT-derived parameters are shown in Table 3 and Figure 2. Maximum choroidal thickness (*p* = 0.136; ES −0.34, 95% CI −0.78 to 0.11), mean choroidal thickness (*p* = 0.097; ES −0.38, 95% CI −0.82 to 0.07), and CVI (*p* = 0.124; ES −0.35, 95% CI −0.79 to 0.10) did not differ significantly between the FMS and control groups.

Within-group Spearman analyses (Table 4) showed no significant correlations between CVI and autonomic symptom burden or clinical scores. In the FMS group, CVI was not correlated with COMPASS-31 (*p* = 0.338), FIQ-R (*p* = 0.667), or CSI (*p* = 0.430). Similarly, no significant correlations were observed in controls (COMPASS-31: *p* = 0.314; FIQ-R: *p* = 0.985; CSI: *p* = 0.268).

In the primary adjusted linear regression model (Model A), group status (FMS vs. control) was not independently associated with CVI (B = −0.013, 95% CI −0.031 to 0.005; *p* = 0.162), whereas age showed a significant negative association with CVI (B = −0.002, 95% CI −0.003 to −0.001; *p* < 0.001). In the sensitivity analysis, additionally adjusted for smoking (Model B), the group coefficient became borderline significant (B = −0.018, 95% CI −0.037 to 0.000; *p* = 0.049), and smoking was positively associated with CVI (B = 0.024, 95% CI 0.004 to 0.044; *p* = 0.019). Further adjustment for educational level (Model C) or occupational status (Model D) did not materially change the results. Within the FMS group, COMPASS-31 total score was not independently associated with CVI after adjustment for age, sex, and BMI (B < 0.001, 95% CI −0.001 to 0.001; *p* = 0.436), while age remained independently and negatively associated with CVI (B = −0.002, 95% CI −0.004 to 0.000; *p* = 0.034).

## 4. Discussion

This cross-sectional study evaluated whether autonomic symptom burden is associated with the CVI in patients with FMS. We found no significant between-group differences in CVI or maximum and mean choroidal thickness, and CVI was not correlated with COMPASS-31, FIQ-R, or CSI within either group. In adjusted analyses, age was independently associated with CVI, whereas group status and autonomic symptom burden were not. Taken together, these findings suggest no clear group-level differences in choroidal structure or vascularity in this cohort, although modest effects cannot be ruled out.

Evidence on retinal and choroidal alterations in FMS remains limited and inconsistent. Ulusoy et al. reported thinner choroidal thickness in patients with FMS than in healthy controls, but found no association with disease severity, WPI, or SSS scores [13]. Sevimli et al. similarly reported reduced choroidal thickness and higher CVI in the FMS group, yet choroidal measures were not related to disease severity, WPI, SSS, or quality-of-life indices [18]. In contrast, Boquete et al. observed no significant between-group differences in choroidal layer parameters [17]. Talu Erten et al. also reported a trend toward thinner choroidal thickness in FMS, without statistical significance, and found no difference in choriocapillaris vessel density compared with controls [16].

Consistent with several prior reports, we found no significant differences between patients with FMS and healthy controls in choroidal thickness or CVI, and CVI was not associated with FIQ-R or CSI scores. Discrepant findings across studies may partly reflect heterogeneity in measurement methods. Ulusoy et al. and Sevimli et al. relied on manual measurements, Talu Erten et al. used device-based automated algorithms, and Boquete et al. applied an artificial intelligence-assisted approach. In the present study, choroidal thickness and CVI were derived using an artificial intelligence–supported automated workflow, which was intended to improve standardisation and reduce observer dependency. Differences in segmentation procedures and the extent of manual input may therefore limit direct comparability of choroidal metrics across studies and contribute to inconsistent results in the literature.

Autonomic symptoms and autonomic dysfunction are commonly reported in FMS [3,5,27,28]. The COMPASS-31, developed to standardise autonomic symptom assessment, is now widely used across clinical populations [21] and has also been validated for assessing autonomic symptom burden in FMS [27,29]. In line with this literature, patients with FMS in our study reported higher COMPASS-31 scores than healthy controls. However, CVI was not associated with overall autonomic symptom burden, and domain-level analyses likewise showed no significant relationships with CVI. This argues against the possibility that an association was simply obscured by aggregation within the total score.

In multivariable models, age was independently and negatively associated with CVI, whereas neither FMS status nor COMPASS-31 total score showed an independent association. In sensitivity analyses, additional adjustment for smoking modestly strengthened the group coefficient, suggesting that lifestyle factors may influence the estimated group effect and should be considered when interpreting null findings. Overall, our results suggest that CVI may be more strongly shaped by fundamental biological determinants, such as age, than by fibromyalgia-related symptom burden. Thus, even when autonomic symptoms are prominent in FMS, this does not necessarily translate into measurable structural differences or a linear relationship within the choroidal vascular compartment.

Sevimli et al. reported higher CVI in patients with FMS than in healthy controls but found no association with disease severity indices; CVI was related only to pain intensity [18]. This pattern may indicate that CVI is more closely linked to vascular reactivity accompanying pain or short-term autonomic fluctuations than to overall symptom burden. While this is biologically plausible given autonomic modulation of choroidal vascular tone, objective autonomic testing in FMS has sometimes shown smaller differences than symptom-based measures [30]. This discrepancy could partly account for the lack of association between COMPASS-31 scores and choroidal parameters in our cohort. Moreover, resting cross-sectional OCT metrics may be relatively insensitive to dynamic vascular regulation, and imaging during sympathetic stimulation may provide a more sensitive assessment of retinal and choroidal vascular reactivity [31].

This study has certain limitations. Firstly, while the cross-sectional design reveals relationships between variables, it limits causal inferences. Second, although the a priori sample size calculation was based on a large assumed effect (Cohen’s d = 1.0), which may be optimistic for OCT outcomes, the achieved sample size provides adequate power mainly for moderate-to-large effects; therefore, the study may be underpowered to detect small-to-medium differences, and non-significant findings should not be interpreted as evidence of no effect. Thirdly, patients using fibromyalgia-specific pharmacological treatment were excluded to reduce confounding with symptom burden and possible autonomic/vascular effects of treatment. While this approach increases internal validity, it may limit the generalisability of the results, particularly to the treated FMS population. Fourthly, autonomic symptoms were assessed based on self-reporting, and autonomic nervous system function was not verified using objective methods. Fifthly, although the refraction values of the groups were similar, ocular axial length, which could affect choroidal parameters, was not assessed. Finally, while there was no significant difference in sex distribution between the groups, the sample was female-dominated, which is consistent with the epidemiology of the disease. This may limit the generalisability of the findings to male FMS patients. Future studies should be designed with larger samples and increased male participant representation.

Despite these limitations, one strength of our study is that it is one of the few investigations to address the relationship between autonomic symptom burden and CVI in FMS simultaneously, using a healthy control group that was matched for age and sex and selected from the same centre. The study also used a standardised OCT assessment with strict ocular and systemic exclusion criteria and an artificial intelligence-assisted measurement approach.

## 5. Conclusions

In this observational cross-sectional study, CVI and maximum/mean choroidal thickness did not differ between patients with FMS and age- and sex-matched healthy controls. Within the FMS group, CVI was not associated with autonomic symptom burden (COMPASS-31) or with fibromyalgia-related clinical burden (CSI and FIQ-R). In multivariable analyses, age was independently associated with CVI, whereas FMS status and COMPASS-31 total score were not. Overall, these findings suggest that CVI, as derived from artificial intelligence-assisted OCT analysis under strict ocular and systemic exclusion criteria, may not reflect self-reported autonomic symptom burden in FMS.

Future studies with larger samples should incorporate objective autonomic function testing and, where feasible, imaging paradigms that capture dynamic vascular regulation to better characterise ocular neurovascular changes in FMS.

## Figures and Tables

**Table 1:** Demographic and baseline characteristics of the study groups.

Variable	Fibromyalgia (*n* = 42)	Control (*n* = 38)	*p* Value
Sex, *n* (%)			0.149 ^c^
Female	38 (90.5)	30 (78.9)	
Male	4 (9.5)	8 (21.1)	
Age (years), mean ± SD	44.79 ± 8.27	43.13 ± 11.35	0.463 ^a^
BMI (kg/m^2^), mean ± SD	26.14 ± 4.67	25.12 ± 3.00	0.128 ^b^
Educational level, *n* (%)			<0.001 *^c^
Primary/Secondary school	22 (52.4)	3 (7.9)	
High school	10 (23.8)	6 (15.8)	
University	10 (23.8)	29 (76.3)	
Occupation, *n* (%)			<0.001 *^c^
Household/retired	23 (54.8)	4 (10.5)	
Desk-based work	7 (16.7)	26 (68.4)	
Physically demanding work	12 (28.6)	8 (21.1)	
Smoking status, *n* (%)			0.052ᶜ
None	26 (61.9)	31 (81.6)	
Current	16 (38.1)	7 (18.4)	

Values are presented as mean ± SD or *n* (%). *p* < 0.05 indicates statistical significance (*). BMI: body mass index; SD: standard deviation. ^a^ Independent samples *t*-test. ^b^ Mann–Whitney U test. ^c^ Pearson chi-square test.

**Table 2:** Symptom severity and autonomic symptom scores in the study groups.

Variable	Fibromyalgia (*n* = 42)	Control (*n* = 38)	*p* Value	ES, 95% CI
FIQ-R	60.17 ± 21.59	13.01 ± 14.32	<0.001 *^b^	0.80 (0.70–0.86)
CSI	58.69 ± 17.28	22.03 ± 14.99	<0.001*^b^	0.76 (0.65–0.84)
COMPASS-31 total score	33.55 ± 15.43	9.49 ± 8.61	<0.001 *^b^	0.73 (0.60–0.82)
Orthostatic intolerance subscore	15.43 ± 10.32	2.63 ± 5.60	<0.001 *^b^	0.63 (0.47–0.74)
Vasomotor subscore	0.99 ± 1.24	0.00 ± 0.00	<0.001 *^b^	0.52 (0.34–0.67)
Secretomotor subscore	4.90 ± 3.08	1.75 ± 2.48	<0.001 *^b^	0.51 (0.32–0.65)
Gastrointestinal subscore	8.48 ± 4.16	3.57 ± 3.02	<0.001 *^b^	0.59 (0.42–0.72)
Bladder subscore	1.30 ± 1.55	0.32 ± 0.57	0.002 *^b^	0.35 (0.14–0.53)
Pupillomotor subscore	2.46 ± 1.21	1.22 ± 0.99	<0.001 *^b^	0.50 (0.31–0.65)

ES: effect size; FIQ-R: Revised Fibromyalgia Impact Questionnaire; COMPASS-31: Composite Autonomic Symptom Score-31; CSI: Central Sensitization Inventory; SD: standard deviation; CI: confidence interval. ^b^ Mann–Whitney U test. ES represents Hedges’ g (bias-corrected standardized mean difference; Fibromyalgia–Control) with 95% confidence intervals. Positive ES values indicate higher scores in the fibromyalgia group. Values are presented as mean ± SD. *p* < 0.05 indicates statistical significance (*).

**Table 3:** Choroidal parameters in the study groups.

Variable	Fibromyalgia (*n* = 42)	Control (*n* = 38)	*p* Value	ES, 95% CI
Maximum choroidal thickness (µm)	365.38 ± 82.45	391.28 ± 69.92	0.136 ^a^	−0.34, (−0.78 to 0.11)
Mean choroidal thickness (µm)	321.11 ± 69.66	346.59 ± 65.37	0.097 ^a^	−0.38, (−0.82 to 0.07)
CVI	0.587 ± 0.049	0.602 ± 0.037	0.124 ^a^	−0.35, (−0.79 to 0.10)

CVI: choroidal vascularity index; SD: standard deviation; ES: effect size; CI: confidence interval. ^a^ Independent samples *t*-test. ES represents Hedges’ g (bias-corrected standardized mean difference; Fibromyalgia–Control) with 95% confidence intervals. Negative ES values indicate lower values in the fibromyalgia group. Values are presented as mean ± SD.

**Table 4:** Association between CVI and autonomic symptom burden and clinical scores.

Variable (with CVI)	Fibromyalgia (*n* = 42)		Control (*n* = 38)	
	r	*p*	r	*p*
COMPASS-31 total score	0.151	0.338	−0.168	0.314
FIQR total score	0.068	0.667	−0.003	0.985
CSI total score	0.125	0.430	−0.184	0.268

CVI: choroidal vascularity index; COMPASS-31: Composite Autonomic Symptom Score-31; FIQR: Revised Fibromyalgia Impact Questionnaire; CSI: Central Sensitization Inventory; r: Spearman’s rank correlation coefficient. *p* values are two-tailed; statistical significance was set at *p* < 0.05.

## Data Availability

The data that support the findings of this study are available from the corresponding author upon reasonable request, subject to applicable ethical and privacy considerations.

## Abbreviations

The following abbreviations are used in this manuscript:

FMS	Fibromyalgia Syndrome
SS-OCT	Swept-Source Optical Coherence Tomography
CVI	Choroidal Vascularity Index
COMPASS-31	Composite Autonomic Symptom Score-31
FIQ-R	Revised Fibromyalgia Impact Questionnaire
CSI	Central Sensitization Inventory
WPI	Widespread Pain Index
SSS	Symptom Severity Scale
BMI	Body Mass Index
